# The mechanism research of itraconazole combined with aspirin in the treatment of vulvovaginal candidiasis through NF-κB signaling pathway

**DOI:** 10.17305/bb.2024.11358

**Published:** 2024-11-22

**Authors:** Tingting Wang, Wenli Feng, Jing Yang, Yan Ma, Zhiqin Xi, Zusha Qiao

**Affiliations:** 1 Department of Dermatovenereology, The Second Hospital of Shanxi Medical University, Shanxi, China

**Keywords:** Vulvovaginal candidiasis, VVC, aspirin, ASP, itraconazole, ITR, synergistic effects, NF-κB signaling pathway

## Abstract

Vulvovaginal candidiasis (VVC) is a common fungal infection caused primarily by *Candida albicans*, characterized by inflammation and discomfort, often requiring effective therapeutic strategies to reduce fungal load and inflammation. This study aimed to explore the therapeutic effects and underlying mechanisms of combining aspirin (ASP) and itraconazole (ITR) in treating VVC through the activation of the NF-κB signaling pathway. Clinical isolates of *C. albicans* were selected, and the M27-A4 microbroth dilution method was used for *in vitro* drug sensitivity testing. A VVC model was established, and after three days of continuous administration, fungal load, inflammatory factors, and pathway protein expression were analyzed using Gram staining, plate counting, glycogen (PAS) staining, ELISA, and qPCR. The results of the *in vitro* drug sensitivity tests revealed that the MIC50 values of ASP and ITR were significantly reduced when the two drugs were combined. In animal experiments, the VVC model group exhibited a substantial vaginal fungal load compared to the blank control group. This was accompanied by elevated levels of inflammatory factors (IL-1β, IL-6, and TNF-α) in serum and vaginal lavage fluid, increased phosphorylation of p65 and IκBα, and upregulation of p65 and IκBα mRNA expression in vaginal tissue. Treatment with the ASP and ITR combination significantly reduced vaginal fungal load, decreased levels of IL-1β, IL-6, and TNF-α, and suppressed the phosphorylation of p65 and IκBα in serum and vaginal lavage fluid. Additionally, the mRNA expression of p65 and IκBα in vaginal tissue was downregulated. These findings suggest that the combination of ASP and ITR is effective in treating VVC. The therapeutic effect may be attributed to the inhibition of IL-1β, IL-6, and TNF-α production by downregulating NF-κB signaling pathway proteins.

## Introduction

Vulvovaginal candidiasis (VVC) is a condition characterized by the colonization of yeast [1]. Its primary symptoms include varying degrees of vaginal itching, burning, increased discharge, difficulty during intercourse, and urinary difficulties, which leads to discomfort and reduced quality of life for patients. Approximately 75% of women experience VVC at least once in their lifetime [2], with over 85% of vaginal fungal infections caused by *Candida albicans* [3]. Azole drugs are widely used for the treatment of VVC due to their good bioavailability and biosecurity, but repeated high-dose and long-term use has led to the development of resistance against the drugs [3], thus limiting the therapeutic value of azole drugs. Drug combinations can not only enhance drug efficacy, reduce adverse drug reactions, and shorten treatment time but also reduce research time and cost, providing new perspectives for VVC treatment. As a commonly used non-steroidal anti-inflammatory drug in clinical practice, aspirin (ASP) exhibits antifungal properties through the inhibition of biofilm formation and modulation of mitochondrial activity [4]. However, the application alone is compromised in terms of safety due to its weak antifungal activity and the necessity for a high dosage. Several studies have shown that it is effective in treating various diseases by inhibiting the activation of the NF-κB pathway [5–7]. It has been reported that *C. albicans* can activate various signaling pathways via NF-κB and c-Fos in vaginal epithelial cells (VECs), playing a key role in coordinating the inflammatory response [8]. In an Italian study, VECs collected from vaginal swabs of healthy women, asymptomatic *C. albicans* carriers, and vaginal candidiasis patients revealed that the transcription factors nuclear factor-κB (NF-κB) and c-Fos-p38 were activated in VECs from symptomatic and asymptomatic pseudomycelia/mycelia carriers. However, they were not activated in asymptomatic yeast carriers [9]. Recent studies have shown that VK2-E6E7 cells infected with *C. albicans* or VVC mice usually trigger the activation of TLRs/NF-κB signaling pathway, inducing an increase in proinflammatory factors, such as TNF-α, IL-1β, and IL-6 [10, 11]. However, no studies have shown whether aspirin exerts antifungal effects by regulating the NF-κB signaling pathway. Our previous research found that the combination of ASP with azole drugs showed a synergistic effect against *C. albicans* in *in vitro* susceptibility tests [12]. Previous *in vitro* susceptibility tests evaluated strains from various sources, such as sputum, urine, feces, and vaginal secretions. The present study aimed to evaluate the *in vitro* antifungal activity of ASP and itraconazole (ITR) against *C. albicans* from the vaginas of VVC patients, investigate the therapeutic effects and mechanisms of the combination of ASP and ITR in VVC treatment through modulation of the NF-κB signaling pathway, and provide an experimental foundation for utilizing combined ASP-ITR therapy in clinical cases resistant to azoles.

## Materials and methods

### Strains

A total of 68 clinical strains of *C. albicans*, were obtained from vaginal secretions of patients diagnosed with VVC at the Obstetrics and Gynecology Department of the Second Hospital of Shanxi Medical University between June and September 2023. These isolates were subsequently purified, identified, and preserved by the Department of Dermatology and Venereology at the Second Clinical College of Shanxi Medical University and the Laboratory of Pathogenic Microorganisms and Immune-Related Skin Diseases Research Center at Shanxi Medical University. The standard *C. albicans* SC5314 strain was purchased from the Fungal Laboratory of the Institute of Dermatology, Chinese Academy of Medical Sciences, and the Fungal Center of the Pathogenic Microorganism (Toxin) Strain Preservation Center of the Chinese Academy of Medical Sciences.

### Preparation of drug storage solutions

ITR powder (1.6 mg) was dissolved in 1 mL of dimethyl sulfoxide (DMSO) for preparation of a stock solution with a concentration of 1600 µg/mL. ASP powder (160 mg) was dissolved in 5 mL of DMSO to prepare a stock solution of 32 mg/mL. Both solutions were stored at −20 ^∘^C.

### Preparation of fungal suspension

The preparation of the fungal suspension involved inoculating *Candida albicans* strains onto yeast extract-peptone-dextrose (YPD) agar medium, followed by incubation at 37 ^∘^C for 48 h. Fresh single colonies were then selected and transferred to YPD liquid medium for further inoculation. This culture was incubated aerobically at 37 ^∘^C with shaking at 220 rpm for 24 h, the culture was centrifuged at 5000 rpm for 2 min to pellet the fungi, which were subsequently washed three times with sterile phosphate-buffered saline (PBS). Finally, the strains were resuspended in YPD liquid medium and diluted to a concentration of 1–5 × 10^3^ cells/mL to prepare the fungal suspension.

### Determination of minimum inhibitory concentration (MIC) under different conditions

The MIC values of ASP, ITR, and their combination against *C. albicans* were determined using the standard M27-A4 microdilution broth method recommended by the Clinical Laboratory and Standards Institute (CLSI). ASP in YPD liquid medium was serially diluted from 16 to 0.25 mg/mL using the doubling dilution method, while ITR was diluted from 16 to 0.0313 µg/mL. YPD liquid medium served as the negative control, and an untreated fungal suspension was used as the positive control. Different concentrations of ITR solutions were added to 96-well plates, followed by the addition of varying concentrations of ASP. After a 48-h incubation at 37 ^∘^C, the MIC was calculated based on *C. albicans* growth in the wells. The MIC was defined as the lowest concentration required to inhibit 50% of *C. albicans* growth [12].

### Determination of fractional inhibitory concentration index (FICI)

The interaction between the two drugs was assessed using FICI. The results were analyzed following the methodology outlined by Bidaud et al. [13], with the calculation formula provided below: FICI ═ (MIC of drug A in combination/MIC of drug A alone) + (MIC of drug B in combination/MIC of drug B alone). The effects of the two antifungal drug combinations were classified as follows: (1) FICI ≤ 0.5 indicates a synergistic effect; (2) 0.5 < FICI ≤ 1 indicates an additive effect; (3) 1 < FICI < 4, no interaction; and (4) FICI ≥ 4.0 indicates an antagonistic effect.

### Breakpoint

According to the CLSI-established M27-A4 broth dilution method, the susceptibility of itraconazole is defined as ≤0.125 µg/mL, while resistance is defined as ≥1 µg/mL.

### Time-growth curve experiment

*C. albicans* strain 72 was selected for the time-growth curve experiment. The strain was cultured overnight, washed with PBS, and adjusted to a suspension of 1–5 × 10^3^ cells/mL in YPD liquid medium. The concentrations of ASP and ITR were determined based on the MIC50 values for the strain. The treatment conditions were as follows.

Control group: 2-mL fungal suspension and 2-mL YPD medium. Single-drug group: 2-mL fungal suspension, 1-mL YPD medium, and 1-mL ITR (2 µg/mL). Drug combination group: 2-mL fungal suspension, 1-mL ITR (2 µg/mL), and 1 mL of varying ASP concentrations (1, 2, 4, 8 mg/mL). The fungal suspension was incubated at 37 ^∘^C with shaking at 220 rpm. Absorbance at 630 nm was measured at 0, 12, 36, and 48 h using a microplate reader. Growth curves were then plotted, and the growth of *C. albicans* strain 72 was recorded at each time point [14].

### Time-kill curve experiment

*C. albicans* strain 72 was selected for the time-kill curve experiment. After 16 h of aerobic incubation at 37 ^∘^C with shaking, 100 µL of the fungal suspension was transferred to 10 mL of fresh YPD liquid medium and cultured for an additional 4 h. The cells were then washed three times with PBS, resuspended in YPD medium, and adjusted to a concentration of 1–5 × 10^3^ CFU/mL using a cell counting plate. The experimental conditions were as follows.

Control group: 2 mL of fungal suspension and 2 mL of YPD medium. Single-drug group: 2 mL of fungal suspension, 1 mL of YPD medium, and 1 mL of ITR (2 µg/mL). Drug combination group: 2 mL of fungal suspension, 1 mL of ITR (2 µg/mL), and 1 mL of ASP (4 mg/mL). The mixtures were incubated aerobically at 30 ^∘^C with shaking (220 rpm) for 0, 12, 24, 36, and 48 h. Following incubation, the fungal suspensions were diluted tenfold and plated onto YPD agar medium. After 48 h of aerobic incubation at 30 ^∘^C, fungal colonies were counted, and the results were plotted as a time-kill curve using log10 CFU/mL values [15].

### VVC mouse model construction and administration

Forty-five 6- to 8-week-old female ICR mice (20 ± 2 g), SPF grade, were purchased from the Experimental Animal Center of Shanxi Medical University [Production License: SCXK (Jin) 2019–0004; Animal Quality Certificate]. All animals were acclimated for seven days under controlled temperature (24 ± 2 ^∘^C) and humidity (50 ± 5%), with free access to food and water in a 12-h light/dark cycle. The mice were randomly divided into five groups: blank control, VVC model, ASP, ITR, and ASP+ITR. Before modeling, each mouse received a subcutaneous injection of 0.1-mL estradiol benzoate (1 mg/mL) into the neck every other day for a total of three injections [10, 14]. For the VVC model, 20 µL of *Candida albicans* suspension (2.5×10^6^/mL) was inoculated into the vagina for five days, while the blank control group received an equivalent volume of PBS. Successful modeling was confirmed by evaluating the general condition of the mice, observing local signs at the vaginal opening, and detecting pseudohyphae and blastospores in a vaginal lavage smear under a microscope. Treatment began the day after successful modeling. Dosages were determined using the mouse-to-human dose conversion factor. The ASP group received 13 mg/kg, the ITR group received 26 mg/kg, and the ASP+ITR group received the same doses of each drug as the single-drug groups. The blank control and VVC model groups received physiological saline by gavage at a dosing volume of 0.2 mL/10 g body weight once daily for three days. Vaginal lavage fluid was collected on the 4th day of treatment for further analysis.

### Observation of general condition and vaginal local signs in the mice

The general condition of the mice in each group was observed daily, focusing on their overall demeanor, activity levels, diet, and fur condition. Additionally, local signs, such as redness, swelling at the vaginal opening, and any secretions, were carefully monitored.

### Microscopic examination of vaginal smears

The morphology of *C. albicans* in vaginal secretions was examined using an optical microscope. On the day following the final treatment, the vaginas were rinsed with 100 µL of PBS, and 5 µL of the vaginal lavage fluid was applied to a coverslip. Gram staining was then conducted to assess the fungal morphology in the vaginal secretions [10].

### Plate counting of *Candida albicans* in vaginal lavage fluid

From each group, 10 µL of vaginal lavage fluid was diluted 1:10 with PBS. The diluted fluid was then spread onto YPD agar plates supplemented with 1% penicillin–streptomycin and incubated at 37 ^∘^C for 48 h. After incubation, the fungal load was quantified by counting colony-forming units (CFU/mL) [10].

### PAS staining for assessing morphological changes in vaginal tissue

The vaginal tissue was fixed in 10% paraformaldehyde for at least 24 h, followed by dehydration, embedding, sectioning, and PAS staining. The pathological conditions of the vaginal mucosa were then examined under a microscope [10].

### ELISA measurement of IL-1, IL-6, TNF-α, p-p65, p65, p-IκBα, and IκBα levels in vaginal lavage fluid and serum

The collected vaginal lavage fluid was centrifuged at 3000 rpm for 15 min. A 10-µL aliquot of the supernatant was then used for incubation, washing, and color development according to the ELISA kit instructions. The absorbance at 450 nm was measured using a microplate reader [10]. Serum samples underwent the same processing steps.

### qPCR measurement of the mRNA expression levels of Rela (p65) and Nfκbia (IκBα) in vaginal tissue

A total of 80 g of vaginal tissue was used to extract total RNA via the TRIzol method. The RNA was then reverse-transcribed into cDNA following the protocol provided with the reverse transcription kit [15]. Real-time quantitative PCR was conducted using the following reaction setup: 0.4 µL of cDNA, 0.4 µL each of upstream and downstream primers, 10 µL of SYBR, and 8.8 µL of ddH2O. The PCR reaction conditions were as follows: pre-denaturation at 95 ^∘^C for 3 min, denaturation at 95 ^∘^C for 5 s, annealing and extension at 60 ^∘^C for 20 s, repeated for 40 cycles. A melting curve was subsequently generated from 60 ^∘^C to 95 ^∘^C. Relative gene expression levels were calculated using the 2^−ΔΔCt^ method, with β-actin serving as the reference gene. Primer sequences for each gene are detailed in Table 1.

**Table 1:** Primer sequences used for qRT-PCR

**Primers**	**Sequence (5′ to 3′)**
*β-actin-*F	ACCGAAGCTCCAATGAATCC
*β-actin-*R	CCGGTGGTTCTACCAGAAGAG
*Rela-*F	ATTGCTGTGCCTACCCGAAAC
*Rela-*R	TTTGAGATCTGCCCTGATGGTAA
*Nfkbia-*F	CAGTGTAGCAGTCTTGACGCAGA
*Nfkbia-*R	GCCAGGTAGCCGTGGATAGA

### Ethical statement

All animal procedures related to this experiment were approved by the Ethics Committee of the Second Hospital of Shanxi Medical University, with the animal ethics number: DW2024063.

### Statistical analysis

SPSS 27.0 software was used for statistical analysis. Measurement data are presented as mean ± standard deviation (SD). A homogeneity test of variance was performed, and if the variance was homogeneous, an independent sample *t*-test was applied for the drug susceptibility test. For multigroup comparisons in animal experiments, a one-way analysis of variance (ANOVA) was conducted, followed by the least significant difference (LSD) test for inter-group comparisons. If variances were unequal, the Dunnett T3 method was used for multigroup comparisons. A *P* value of <0.05 was considered statistically significant. Graphs were generated using GraphPad 9.5 and Excel software.

## Results

### Comparison of drug sensitivity of ASP and ITR alone and in combination

The MIC50 values of ASP monotherapy, ITR monotherapy, and their combination against clinical *C. albicans* isolates were determined using the standard microdilution broth method (CLSI M27-A4). The specific MIC50 values are shown in Table 2. The results indicate that the MIC50 of ASP when used alone ranged from 250–2000 µg/mL, while the MIC50 of ITR alone ranged from 0.0313–16 µg/mL. Among the 68 clinical isolates, 34 (50%) were resistant to ITR. When ASP was combined with ITR, the MIC50 of ASP decreased to 250–1000 µg/mL, and the MIC50 of ITR decreased to 0.0313–2 µg/mL. These findings suggest that the MIC50 values of both ASP and ITR against *C. albicans* were reduced when the drugs were used in combination compared to monotherapy.

**Table 2:** MIC50 values of ASP and ITR

		**MIC50(µg/mL)**			
		**ASP**		**ITR**	
**Strain**	**FICI**	**Alone**	**Combination**	**Alone**	**Combination**
2	0.75	2000	1000	0.125	0.0313
3	0.1875	2000	250	2	0.125
5	0.2656	2000	500	4	0.0625
8	0.3754	2000	250	0.125	0.0313
9	0.1289	2000	250	8	0.0313
11	0.2539	2000	500	8	0.0313
12	0.375	2000	250	0.125	0.0313
13	0.2578	1000	250	4	0.0313
15	0.625	2000	250	0.0625	0.0313
16	0.625	2000	250	0.0625	0.0313
17	1.125	2000	250	0.0313	0.0313
20	1.5	2000	1000	0.0313	0.0313
21	0.2539	2000	500	8	0.0313
22	1.25	4000	1000	0.5	0.5
24	1.25	1000	250	0.0313	0.0313
26	0.5626	1000	500	0.5	0.0313
28	1.25	1000	250	0.0313	0.0313
32	0.1875	2000	250	0.5	0.0313
33	0.7504	1000	500	0.125	0.0313
34	1	2000	1000	0.0625	0.0313
35	1.25	2000	250	0.0313	0.0313
39	0.75	2000	500	0.0625	0.0313
41	0.502	1000	500	16	0.0313
42	1.25	1000	250	0.0313	0.0313
43	0.2813	2000	500	1	0.0313
44	1	2000	1000	0.0625	0.0313
45	0.1563	2000	250	1	0.0313
46	0.2656	2000	500	2	0.0313
47	0.502	1000	500	16	0.0313
48	0.2578	2000	500	4	0.0313
49	0.75	2000	500	0.0625	0.0313
50	1	2000	1000	0.125	0.0625
51	0.1875	2000	250	1	0.0625
52	0.625	2000	250	0.0625	0.0313
53	1.25	2000	250	0.0313	0.0313
54	0.5	2000	500	0.125	0.0313
55	0.5313	1000	500	0.0313	0.0313
57	0.2656	2000	500	4	0.0625
58	0.625	2000	250	0.125	0.0625
59	0.5078	1000	500	4	0.0313
61	0.1328	2000	250	4	0.0313
62	0.3125	2000	500	1	0.0625
63	0.75	2000	500	0.0625	0.0313
64	1.25	1000	250	0.0313	0.0313
65	0.2656	2000	500	2	0.0313
66	0.2539	2000	500	8	0.0313
69	0.2578	2000	500	8	0.0625
70	1.0078	250	250	4	0.0313
71	0.1328	2000	250	8	0.0625
72	0.1328	2000	250	4	0.0313
73	0.2539	2000	500	8	0.0313
74	0.25	2000	250	2	0.25
75	0.625	2000	250	0.125	0.0625
76	0.5039	2000	1000	8	0.0313
77	1.25	2000	250	0.0313	0.0313
78	1.25	2000	250	0.0313	0.0313
79	0.375	2000	250	8	2
80	0.75	2000	500	0.125	0.0625
82	1.125	2000	250	0.0625	0.0625
83	0.75	2000	500	0.25	0.125
84	1.125	2000	250	0.0313	0.0313
86	0.3125	1000	250	8	0.5
87	0.1328	2000	250	8	0.0625
88	0.2539	2000	500	16	0.0625
89	0.75	2000	250	1	0.5
90	0.5039	1000	500	8	0.0313
91	0.2539	2000	500	8	0.0313
93	0.2539	2000	500	8	0.0313

### Interaction of ASP and ITR against *Candida albicans*

The FICI was calculated to assess the interaction between ASP and ITR on the sensitivity of *Candida albicans*. Synergistic effects were observed in 31 strains (FICI ≤ 0.5), additive effects in 23 strains (0.5 < FICI ≤ 1), no effect in 14 strains (1 < FICI < 4), and antagonistic effects in none of the strains (FICI ≥ 4). The enhancement efficiency of ASP on ITR was determined to be 45.59% (Table 3).

### Time-growth curve analysis

**Figure 1.**
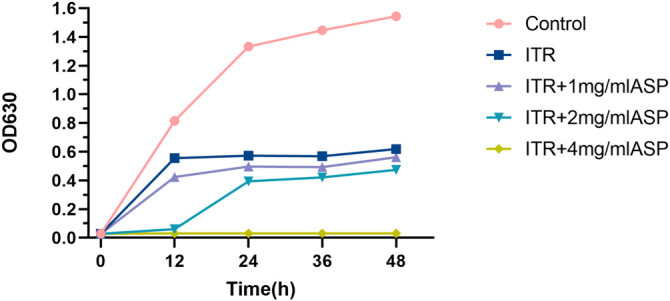
**Time-growth curves of ASP alone, ITR alone, and their combination.** ASP: Aspirin; ITR: Itraconazole.

Time-growth curves were generated for *C. albicans* strain 72 treated with ITR alone or in combination with varying concentrations of ASP. As shown in Figure 1, consistent with the drug susceptibility testing results, ITR (2 µg/mL) alone inhibited the growth of *C. albicans* strain 72. Notably, the combination of ASP and ITR further suppressed the growth of *C. albicans* 72, demonstrating a stronger synergistic inhibitory effect at 4 mg/mL ASP. Based on these findings, 2 µg/mL ITR and 4 mg/mL ASP were selected for subsequent time-kill experiments.

### Time-kill curve analysis

**Figure 2.**
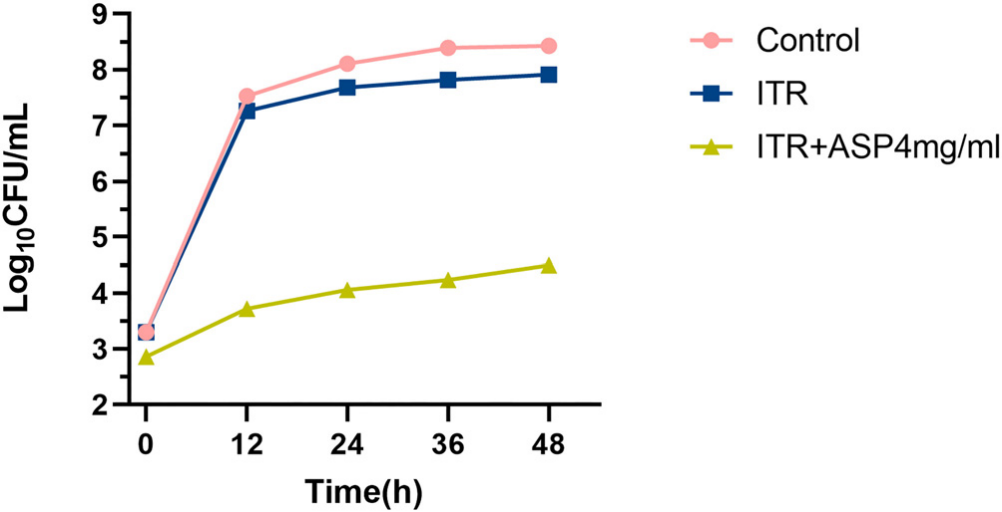
**Time-kill curves of ASP alone, ITR alone, and their combination.** ASP: Aspirin; ITR: Itraconazole.

After treatment with ITR alone, ASP alone, and their combination, the total number of colonies was determined at each time point to evaluate the synergistic inhibitory effect of the drugs on *C. albicans* strain 72. At 12, 24, 36, and 48 h of incubation, both ITR alone and in combination with ASP inhibited *C. albicans* growth. The colony counts in the ASP+ITR group were significantly lower than those in the ITR group (Figure 2), demonstrating that the ASP and ITR combination exerted a more pronounced inhibitory effect on *C. albicans* compared to ITR alone.

### Effects of the ASP and ITR combination on the general condition and vaginal local signs in VVC mice

**Figure 3.**
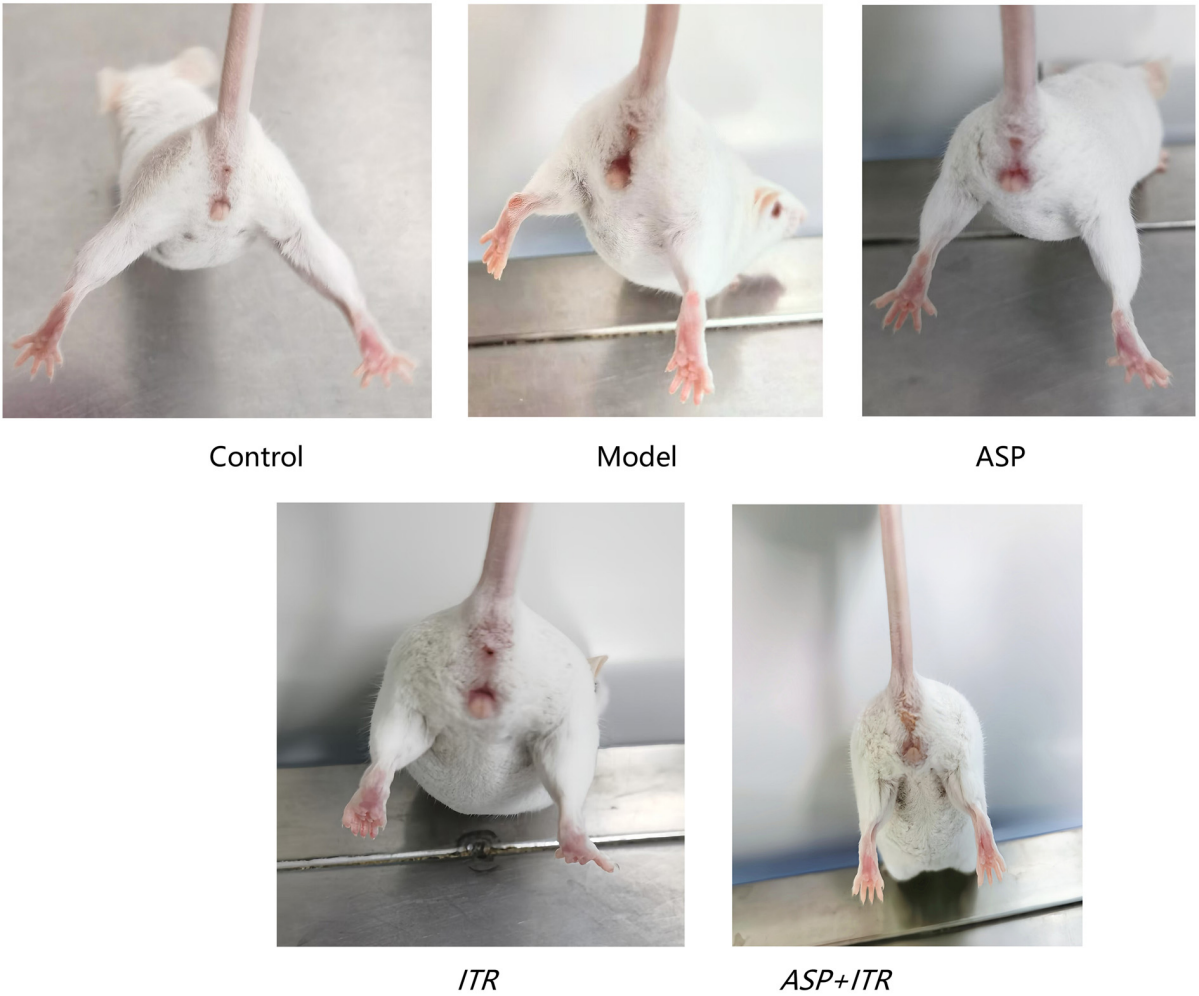
**Effects of ASP and ITR on the appearance of mouse vagina.** ASP: Aspirin; ITR: Itraconazole.

Mice in the blank control group exhibited normal activities without any significant changes. Following modeling, the mice in the model group displayed redness and swelling of the vulva, along with a white viscous discharge and reduced activity levels. After drug treatment, the ASP and ITR groups showed improvements, with reduced vaginal symptoms and discharge, although some redness and swelling at the vaginal opening persisted. In the ASP+ITR group, no redness, swelling, or discharge was observed in the vagina. These findings suggest that both ASP and ITR have therapeutic effects on VVC, with their combined use showing superior efficacy (Figure 3).

**Table 3:** Interaction between ASP and ITR

	**ASP combined with ITR**
Additive (0.5<FICI≤1)	23
No interaction (I<FICI<4)	14
Antagonistic (FICI≥4)	0
Sensitization rate	45.59%

### Effects of the ASP and ITR combination on the morphology of *C. albicans* in vaginal lavage fluid of VVC mice

**Figure 4.**
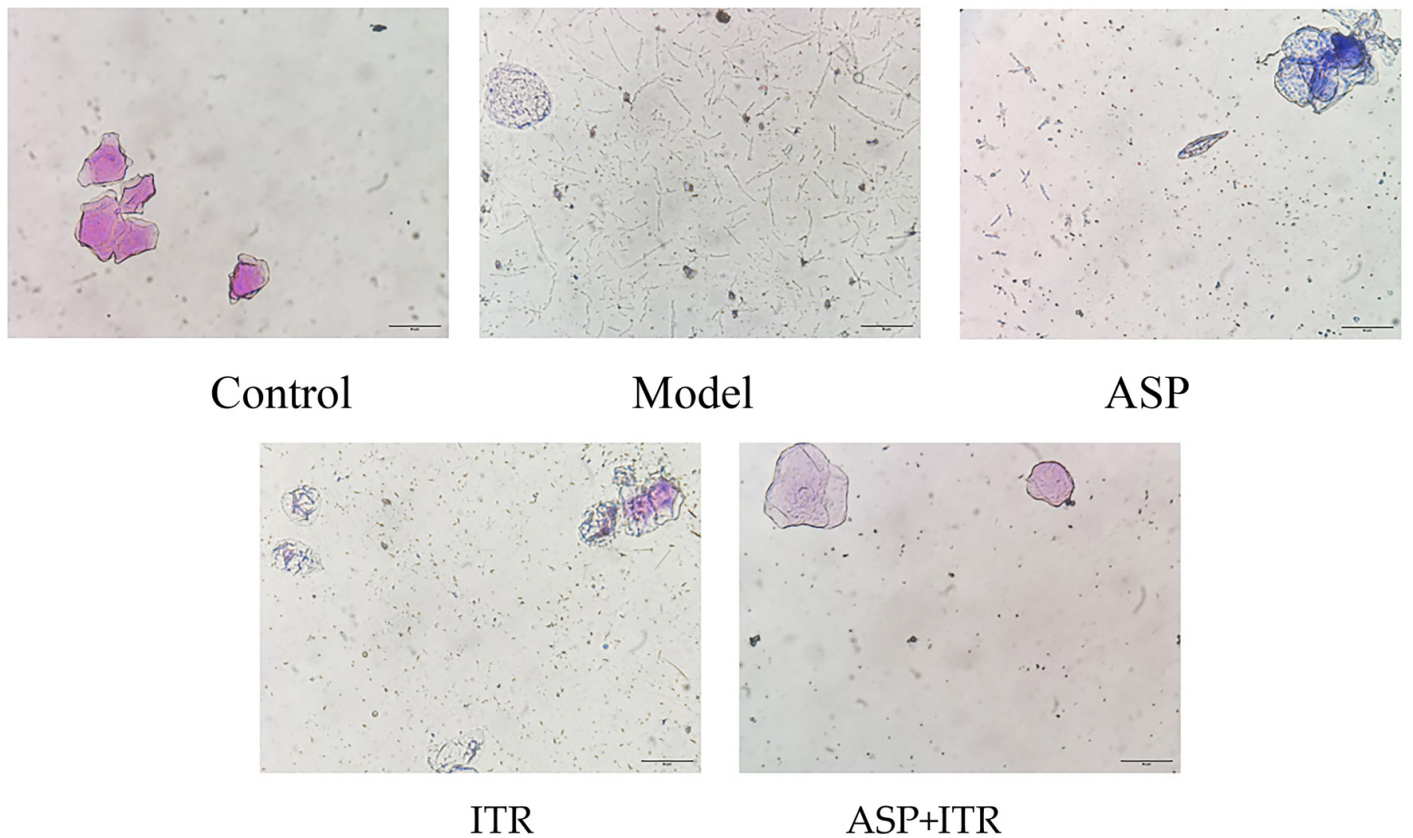
**Effects of ASP and ITR on the morphology of *Candida albicans* hyphae in mouse vaginal secretions (HE staining, ×400) Scale bar: 50 µm.** ASP: Aspirin; ITR: Itraconazole.

Compared to the blank control group, the vaginal lavage fluid of the VVC model group mice contained a large number of elongated hyphae and yeast-like cells. In contrast, the ASP and ITR groups showed a reduction in pseudohyphae with significantly shorter lengths, although the overall fungal load was not substantially reduced. Notably, in the ASP+ITR group, no pseudohyphae were detected in the vaginal lavage fluid, and the fungal load was significantly decreased. Detached vaginal epithelial cells and clue cells were observed across all groups (Figure 4).

### Effects of the ASP and ITR combination on the fungal load of *C. albicans* in vaginal lavage fluid of VVC mice

**Figure 5.**
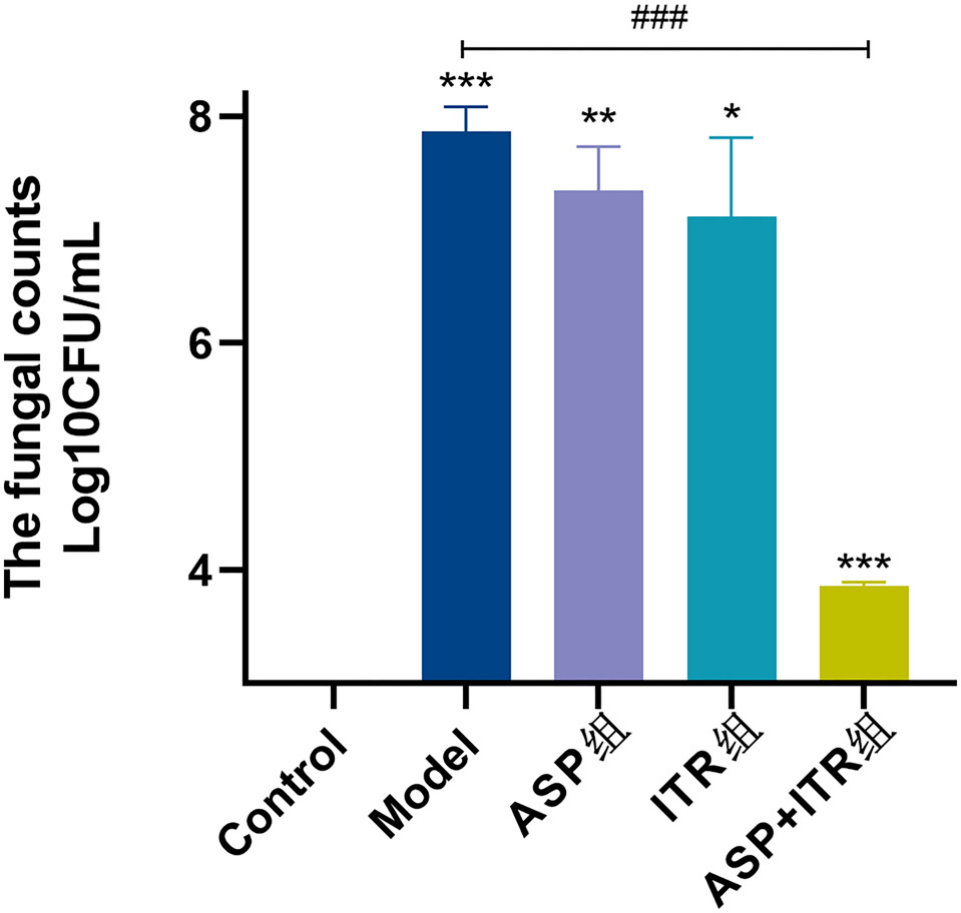
**Effects of ASP and ITR on the number of fungi in mouse vaginal lavage fluid (*n* ═ 3).** Note: Compared with the blank control group *P < 0.05, **P < 0.01, ***P < 0.001; compared with the model group ^#^*P* < 0.05, ^##^*P* < 0.01, ^###^*P* < 0.001. ASP: Aspirin; ITR: Itraconazole.

As shown in Figure 5, *C. albicans* was not detected in the vaginal cavities of mice in the blank control group. In contrast, a significant amount of *C. albicans* was present in the vaginal cavities of the model group mice, with the difference being statistically significant difference (*P* < 0.001). The number of *C. albicans* in the vaginal cavities of mice in the ASP, ITR, and ASP+ITR groups decreased to varying degrees. However, the reductions in the ASP and ITR groups were not substantial and did not reach statistical significance. In comparison, the ASP+ITR group showed a significance (*P* < 0.001).

### Effects of the ASP and ITR combination on the histopathology of vaginal tissue from VVC mice

**Figure 6.**
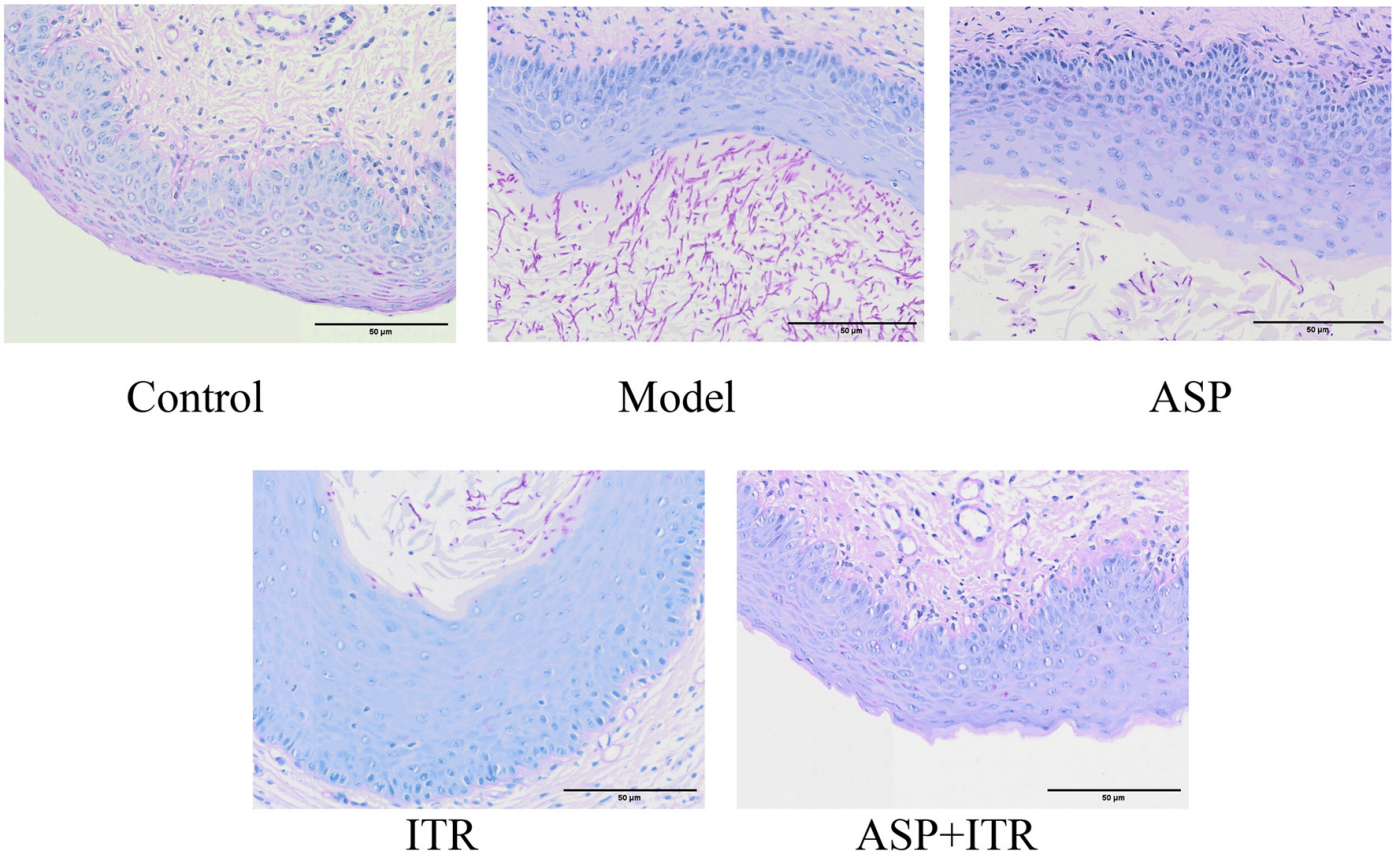
**Effects of ASP and ITR on the histopathology of vaginal tissue in mice (PAS staining, ×400).** Scale bar: 50 µm. ASP: Aspirin; ITR: Itraconazole.

Histopathological sections stained with PAS reveal the infection status of *C. albicans* and the glycogen distribution in vaginal tissue following treatment. The results indicated that the vaginal mucosa of mice in the blank control group remained intact, with clearly defined structural layers and visible glycogen in the keratinized layer of the vaginal mucosal epithelium; no spores or hyphae were detected. In contrast, the model group exhibited the complete disappearance of glycogen in the keratinized layer of the vaginal mucosa, along with the presence of numerous spores and hyphae in the vaginal mucosal epithelium. Post-treatment with ASP or ITR, the number of spores and hyphae in the vaginal mucosal epithelium was markedly reduced, although glycogen was absent in the keratinized layer. Notably, in the combined treatment group, the reduction in spores and hyphae was even more pronounced, and glycogen levels in the keratinized layer were higher compared to the single-drug treatment groups (Figure 6).

### Effects of the ASP and ITR combination on IL-1, IL-6, and TNF-α levels in vaginal lavage fluid and serum of VVC mice

**Figure 7.**
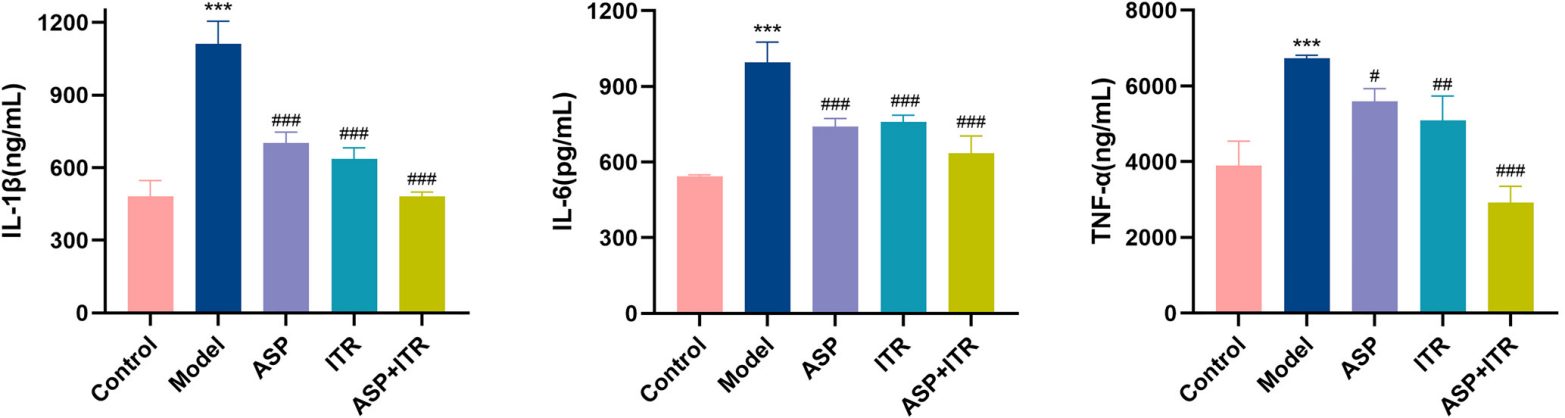
**Effects of ASP and ITR on IL-1, IL-6, and TNF-*α* levels in vaginal lavage fluid of VVC mice (*n* ═ 3).** Note: Compared with the blank control group, *P < 0.05, **P < 0.01, ***P < 0.001; compared with the model group, ^#^*P* < 0.05, ^##^*P* < 0.01, ^###^*P* < 0.001. ASP: Aspirin; ITR: Itraconazole; VVC: Vulvovaginal candidiasis.

**Figure 8.**
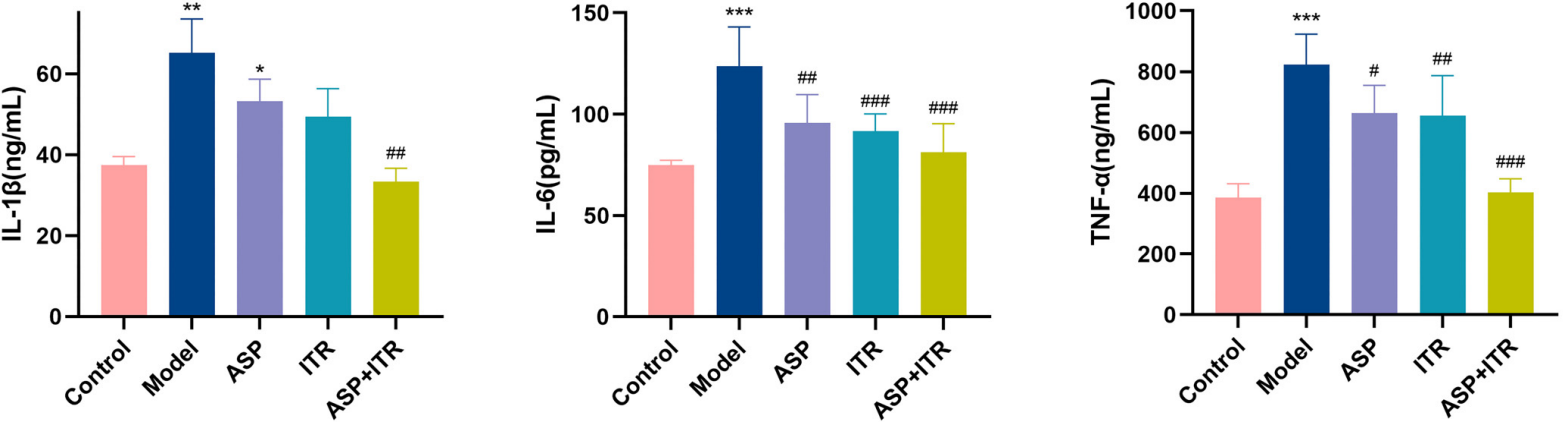
**Effects of ASP and ITR on IL-1, IL-6, and TNF-α levels in serum of VVC mice (*n* ═ 5).** Note: Compared with the blank control group, *P < 0.05, **P < 0.01, ***P < 0.001; compared with the model group, ^#^*P* < 0.05, ^##^*P* < 0.01, ^###^*P* < 0.001. ASP: Aspirin; ITR: Itraconazole; VVC: Vulvovaginal candidiasis.

Compared to the Control group, levels of IL-1β in both vaginal lavage fluid and serum were significantly elevated in the Model group (*P* < 0.01, *P* < 0.001). In contrast, the ASP group, ITR group, and ASP+ITR group showed a notable reduction in IL-1β levels in both vaginal lavage fluid and serum in the ASP group and the ASP+ITR group 0.05, *P* < 0.01). Significant differences among all groups were observed for IL-1β levels in vaginal lavage 0.001). The expression of IL-6 followed a similar pattern. IL-6 levels were significantly increased in the Model group compared to the Control group (*P* < 0.001). However, IL-6 levels decreased significantly in the ASP, ITR, and ASP+ITR groups when compared to the Model group serum samples (*P* < 0.01, *P* < 0.001). For TNF-α, its levels were higher in the Model group than in the Control group (*P* < 0.001), but lower in the ASP, ITR, and ASP+ITR groups P IL-1β, IL-6, and TNF-α levels in both vaginal lavage fluid and serum were more pronounced in the ASP+ITR group compared to the ASP and ITR groups (Figures 7 and 8).

### Effects of the ASP and ITR combination on the expression levels of NF-κB signaling pathway proteins in vaginal lavage fluid and sera of VVC mice

**Figure 9.**
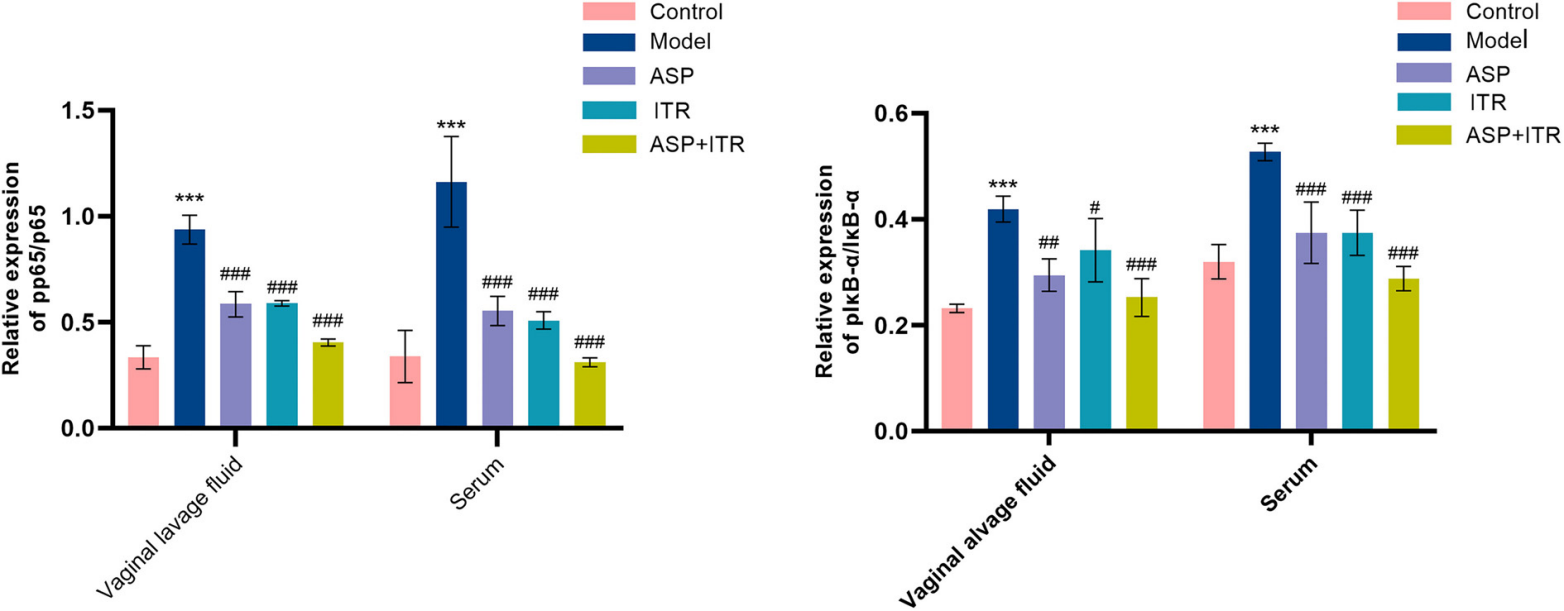
**Effects of ASP and ITR on p-p65/p65 and p-IκBα/IκBα ratios in vaginal lavage fluid and serum of VVC mice (vaginal lavage fluid, *n* ═ 3; serum, *n* ═ 5).** Note: Compared with the blank control group, *P < 0.05, **P < 0.01, ***P < 0.001; compared with the model group, ^#^*P* < 0.05, ^##^*P* < 0.01, ^###^*P* < 0.001. ASP: Aspirin; ITR: Itraconazole; VVC: Vulvovaginal candidiasis.

Compared to the control group, both the ratio of P-P65/p65 in vaginal lavage fluid and serum was significantly increased in the model group (*P* < 0.001, *P* < 0.001). However, in mice treated with ASP, ITR or ASP+ITR, this ratio decreased to varying degrees compared to the model group (*P* < 0.05, *P* < 0.05, *P* < 0.01) in both vaginal lavage fluid and serum (*P* < 0.001), showing a statistically significant difference between groups. The p-IκBα/IκBα ratio exhibited a similar trend as that of p-P65/p65; it significantly increased compared to the Control group but significantly decreased in all treatment groups compared to the Model group for both vaginal lavage fluid and serum ratios (*P* < 0.01, *P* < 0.05, *P* < 0.001). Notably, ASP+ITR treatment resulted in a significant decrease of these ratios (Figure 9).

### Effects of the ASP and ITR combination on the mRNA Levels of NF-κB signaling pathway components in the vaginal tissue of VVC mice

**Figure 10.**
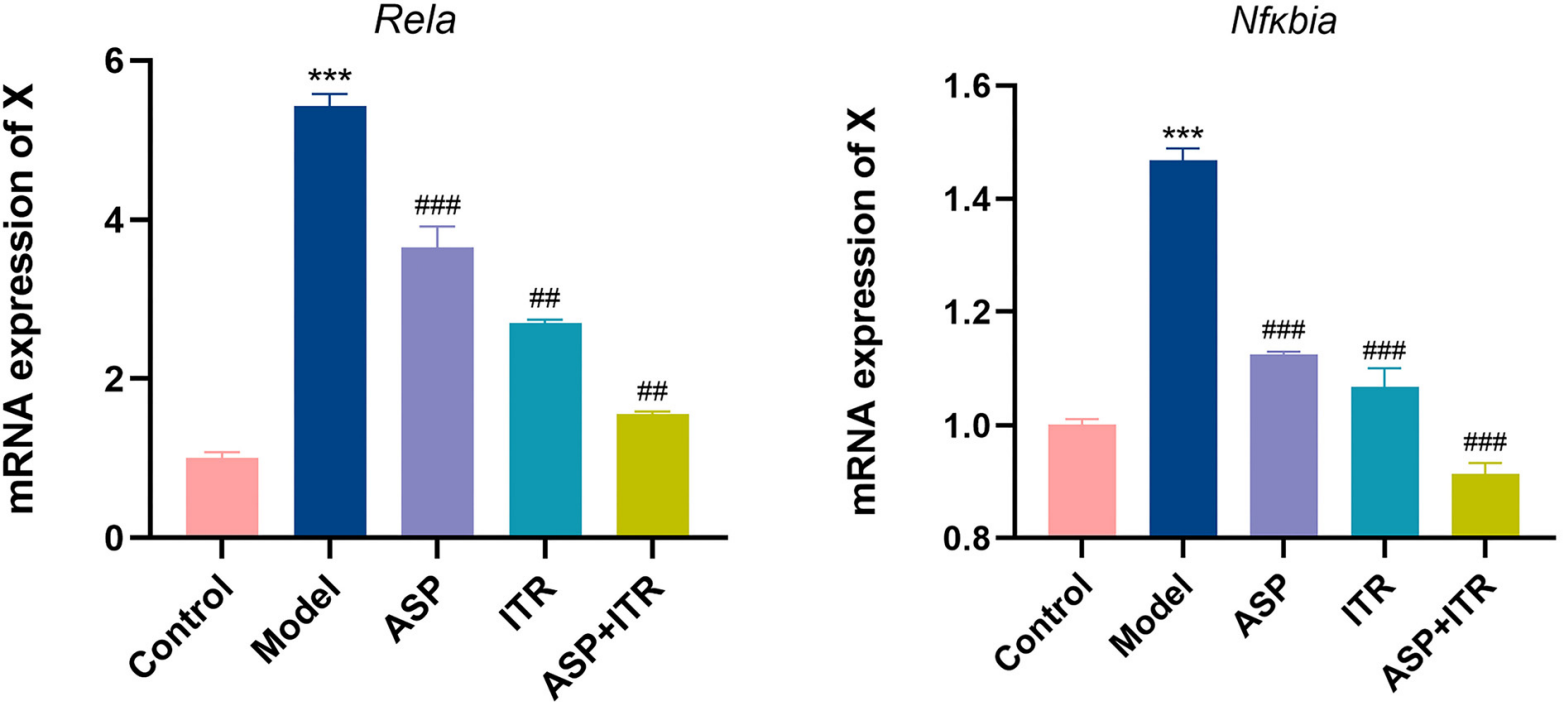
**Effects of ASP and ITR on mRNA expression levels of p65 and IκBα in the vaginal tissue of VVC mice (*n* ═ 3).** Note: Compared with the blank control group, *P < 0.05, **P < 0.01, ***P < 0.001; compared with the model group, ^#^*P* < 0.05, ^##^*P* < 0.01, ^###^*P* < 0.001. ASP: Aspirin; ITR: Itraconazole; VVC: Vulvovaginal candidiasis.

The qPCR results demonstrated that, compared to the control group, mice in the model group exhibited significantly increased mRNA expression levels of Rela (p65) and Nfκbia (IκBα) in vaginal tissue (*P* < 0.001). However, treatment with ASP, ITR, or their combination significantly reduced the mRNA levels of p65 and IκBα in the vaginal tissue, the ASP+ITR combination produced a more substantial decrease in the mRNA expression levels of p65 and IκBα than either the ASP or ITR treatments alone (Figure 10).

## Discussion

Azole drugs are currently the first-line treatment for vulvovaginal candidiasis (VVC); however, increasing resistance to these drugs has become a significant concern. The mechanisms underlying azole resistance primarily include enhanced efflux pump activity, mutations in the ERG11 gene, metabolic changes, and biofilm formation [16, 17]. Studies have demonstrated that, in outpatient cases of refractory or recurrent VVC, antifungal susceptibility tests reveal that *Candida albicans* strains isolated from patients with recurrent VVC (RVVC) consistently exhibit stable resistance to fluconazole. This resistance persists even after the discontinuation of fluconazole treatment [18]. As a result, exploring novel therapeutic options is essential to improving VVC treatment outcomes and addressing resistance in *C. albicans*. Currently, combination drug therapies have become a focus of research in combating Candida resistance. Previous studies suggest that ASP can increase the susceptibility of azole-resistant strains in *in vitro* susceptibility tests, further inhibiting Candida growth [12, 19, 20]. This study not only confirms the synergistic antifungal effects of combining ASP with ITR against *C. albicans* isolated from VVC patients but also demonstrates significant therapeutic efficacy in VVC mouse models. Specifically, the MIC50 values for ASP and ITR decreased from 250–2000 µg/mL to 250–1000 µg/mL, while the MIC50 value for ITR alone was reduced from 0.0313–16 µg/mL to 0.0313–2 µg/mL. Fractional inhibitory concentration index (FICI) determinations revealed that ASP enhanced ITR efficacy by 45.59%. Time-growth and time-kill curve analyses of *C. albicans* further demonstrated the synergistic effect of ASP on ITR. In animal experiments, VVC mice treated with the ASP–ITR combination showed significant improvements, including reduced vaginal redness and swelling, increased secretion, lower fungal loads in vaginal secretions, and inhibited hyphal morphology conversion. Histopathological examination of vaginal mucosa revealed alleviated fungal infection, alongside the disappearance of glycogen deposits. These findings suggest that the ASP-ITR combination has a clear synergistic therapeutic effect in VVC mouse models.

The pathogenicity of *C. albicans* in VVC is closely linked to its virulence factors, including adhesins, hydrolytic enzymes, biofilm formation, and dimorphism, as well as the host immune responses that contribute to mucosal damage [21]. VVC is an inflammatory disease of the vaginal mucosa caused by *C. albicans* infection, where the adhesion of *C. albicans* to vaginal epithelial cells represents the initial step in disease development [22]. Since the vaginal mucosal surface is constantly exposed to *C. albicans*, vaginal epithelial cells play a crucial role in responding to opportunistic microbes when they become pathogenic, initiating an appropriate innate immune response [23]. Microbial adhesins interact with host pattern recognition receptors (PRRs), particularly TLR2 and TLR4, on epithelial cell surfaces [24]. This interaction triggers the activation of multiple signaling pathways—most notably the NF-κB pathway—via specific adapter molecules, ubiquitin ligases, and protein kinases. These pathways transmit signals from the cell surface to the nucleus, inducing proinflammatory factor expression and driving the inflammatory response [25]. Research has shown that high-glucose conditions promote NF-κB pathway activation, resulting in increased release of downstream pro-inflammatory factors, such as TNF, IL-1, IL-6, and IL-18. These factors recruit immune cells like monocytes, neutrophils, lymphocytes, and macrophages to affected tissues [26]. Additionally, studies indicate that inflammatory cytokines, including TNF, IL-1, and IL-6, accumulate in inflamed tissues, leading to NF-κB phosphorylation and further activation of this pathway [27]. Consistent with these findings, the current study observed NF-κB pathway activation and a significant release of inflammatory cytokines—such as TNF-α, IL-1, and IL-6—during *C. albicans*-induced VVC. Compared to the blank control group, inflammatory cytokine levels were markedly elevated in the VVC mouse model. However, combined treatment with ASP and ITR significantly reduced the levels of IL-1β, IL-6, and TNF-α in the vaginal secretions of VVC mice.

In the classical NF-κB pathway, the p50/p65 dimer binds to IκB and remains inhibited. Proinflammatory factors and LPS stimulate the activation of the IKK complex (comprising IKKα, IKKβ, and IKKγ/NEMO), which phosphorylates IκB proteins. This phosphorylation triggers the ubiquitination of IκB, leading to its lysosomal degradation and the release of p50/p65. The activated p50/p65 dimer is further phosphorylated and translocated to the nucleus, where, either alone or in conjunction with other transcription factors, such as AP-1, Ets, or Stat, it induces the expression of target genes [25]. In the present study, compared to the blank control group, the phosphorylation levels of p65 and IκBα proteins, as well as the mRNA levels of p65 and IκBα, were increased in the VVC mouse model. Treatment with ASP or ITR reduced the expression of these factors to varying degrees, while their combination demonstrated a more pronounced reduction. These findings suggest that the combination of ASP and ITR exerts therapeutic effects on VVC by inhibiting p65 and IκBα protein phosphorylation and modulating the NF-κB pathway. The combination of ASP and ITR represents a novel therapeutic approach to VVC treatment. *In vitro* experiments revealed that the combined MIC50 value of ASP and ITR was significantly lower than that of either ASP or ITR alone. *In vivo* experiments further demonstrated that the combined administration of ASP and ITR significantly reduced vaginal fungal loads in mice, while also decreasing proinflammatory cytokines, including IL-1β, IL-6, and TNF-α, in vaginal tissues compared to single-agent treatments. Mechanistic studies revealed that the synergistic activity of ASP and ITR against *C. albicans* was associated with the inhibition of the NF-κB signaling pathway. This study highlights the synergistic effects of ASP and ITR in the treatment of VVC. The combination appears to mitigate inflammatory damage to the vaginal mucosa by downregulating protein expression in the NF-κB signaling pathway. Despite these findings, the study has limitations, including a small experimental sample size, biological variability among individual mice, and potential experimental errors, all of which may impact the results. Additionally, further investigations are needed to resolve outstanding issues related to the mouse VVC model before clinical application. These include determining optimal dosages and treatment ratios, as well as evaluating drug safety and potential side effects. Moreover, it remains unclear whether the drug combination influences other signaling pathways besides NF-κB. Further research is needed to identify upstream and downstream factors within the NF-κB pathway that regulate immune responses. The completion of these investigations will provide valuable insights into the clinical application of ASP and ITR for the treatment of VVC.

## Conclusion

In conclusion, the combined application of ASP and ITR has a clear impact on VVC. Animal experiments suggest that this combination may inhibit the production of inflammatory mediators such as IL-1β, IL-6, and TNF-α by downregulating protein expression within the NF-κB signaling pathway. This effect could play a significant role in the effective management of VVC.

## References

[1] Nyirjesy P, Brookhart C, Lazenby G, Schwebke J, Sobel JD (2022). Vulvovaginal candidiasis: a review of the evidence for the 2021 centers for disease control and prevention of sexually transmitted infections treatment guidelines. Clin Infect Dis.

[2] Esfahani A, Omran AN, Salehi Z, Shams-Ghahfarokhi M, Ghane M, Eybpoosh S (2022). Molecular epidemiology, antifungal susceptibility, and ERG11 gene mutation of Candida species isolated from vulvovaginal candidiasis: comparison between recurrent and non-recurrent infections. Microb Pathog.

[3] Mollazadeh-Narestan Z, Yavarikia P, Homayouni-Rad A, Samadi Kafil H, Mohammad-Alizadeh-Charandabi S, Gholizadeh P (2023). Comparing the effect of probiotic and fluconazole on treatment and recurrence of vulvovaginal candidiasis: a triple-blinded randomized controlled trial. Probiotics Antimicrob Proteins.

[4] Babaei F, Mirzababaei M, Tavakkoli A, Nassiri-Asl M, Hosseinzadeh H (2024). Can nonsteroidal anti-inflammatory drugs (NSAIDs) be repurposed for fungal infection?. Naunyn Schmiedebergs Arch Pharmacol.

[5] Chen CM, Tung YT, Wei CH, Lee PY, Chen W (2020). Anti-Inflammatory and reactive oxygen species suppression through aspirin pretreatment to treat hyperoxia-induced acute lung injury in NF-κB-Luciferase inducible transgenic mice. Antioxidants (Basel).

[6] Wu L, Luo Z, Liu Y, Jia L, Jiang Y, Du J (2019). Aspirin inhibits RANKL-induced osteoclast differentiation in dendritic cells by suppressing NF-κB and NFATc1 activation. Stem Cell Res Ther.

[7] Chang Y, Kong K, Tong Z, Qiao H, Hu Y, Xia R (2023). Aspirin prevents estrogen deficiency-induced bone loss by inhibiting osteoclastogenesis and promoting osteogenesis. J Orthop Surg Res.

[8] Moyes DL, Murciano C, Runglall M, Islam A, Thavaraj S, Naglik JR (2011). Candida albicans yeast and hyphae are discriminated by MAPK signaling in vaginal epithelial cells. PLoS One.

[9] Roselletti E, Perito S, Sabbatini S, Monari C, Vecchiarelli A (2019). Vaginal epithelial cells discriminate between yeast and hyphae of candida albicans in women who are colonized or have vaginal candidiasis. J Infect Dis.

[10] Feng X, Zhang H, Hu K, Shi G, Wu D, Shao J (2024). Longdan Xiegan decoction ameliorates vulvovaginal candidiasis by inhibiting the NLRP3 inflammasome via the Toll-like receptor /MyD88 pathway. J Ethnopharmacol.

[11] Li W, Yin Y, Li T, Wang Y, Shi W (2024). Licochalcone a protects vaginal epithelial cells against candida albicans infection via the TLR4/NF-κB signaling pathway. J Microbiol.

[12] Feng W, Yang J, Ma Y, Xi Z, Ji Y, Ren Q (2021). Cotreatment with aspirin and azole drugs increases sensitivity of Candida albicans in vitro. Infect Drug Resist.

[13] Bidaud AL, Schwarz P, Chowdhary A, Dannaoui E (2022). In vitro antifungal combination of terbinafine with itraconazole against isolates of trichophyton species. Antimicrob Agents Chemother.

[14] He Y, Tang R, Deng J, Cai T, He P, Wu J (2022). Effects of oestrogen on vulvovaginal candidosis. Mycoses.

[15] Yu G, Yu H, Yang Q, Wang J, Fan H, Liu G (2022). Vibrio harveyi infections induce production of proinflammatory cytokines in murine peritoneal macrophages via activation of p38 MAPK and NF-κB pathways, but reversed by PI3K/AKT pathways. Dev Comp Immunol.

[16] Harley BK, Neglo D, Tawiah P, Pipim MA, Mireku-Gyimah NA, Tettey CO (2021). Bioactive triterpenoids from Solanum torvum fruits with antifungal, resistance modulatory and anti-biofilm formation activities against fluconazole-resistant candida albicans strains. PLos One.

[17] Sobel JD, Sobel R (2018). Current treatment options for vulvovaginal candidiasis caused by azole-resistant Candida species. Expert Opin Pharmacother.

[18] Sobel JD, Sebastian S, Boikov DA (2023). A longitudinal study on fluconazole resistance in Candida albicans vaginal isolates. Mycoses.

[19] Ahangarkani F, Khodavaisy S, Mahmoudi S, Shokohi T, Rezai MS, Fakhim H (2019). Indifferent effect of nonsteroidal anti-inflammatory drugs (NSAIDs) combined with fluconazole against multidrug-resistant Candida auris. Curr Med Mycol.

[20] Król J, Nawrot U, Bartoszewicz M (2018). Anti-candidal activity of selected analgesic drugs used alone and in combination with fluconazole, itraconazole, voriconazole, posaconazole and isavuconazole. J Mycol Med.

[21] Czechowicz P, Nowicka J, Gościniak G (2022). Virulence factors of *Candida* spp. and host immune response important in the pathogenesis of vulvovaginal candidiasis. Int J Mol Sci.

[22] Zhao T, Zhang K, Shi G, Ma K, Wang B, Shao J (2022). Berberine inhibits the adhesion of candida albicans to vaginal epithelial cells. Front Pharmacol.

[23] Naglik JR, Richardson JP, Moyes DL (2014). Candida albicans pathogenicity and epithelial immunity. PLoS Pathog.

[24] Pivarcsi A, Nagy I, Koreck A, Kis K, Kenderessy-Szabo A, Szell M (2005). Microbial compounds induce the expression of pro-inflammatory cytokines, chemokines and human beta-defensin-2 in vaginal epithelial cells. Microbes Infect.

[25] Yu H, Lin L, Zhang Z, Zhang H, Hu H (2020). Targeting NF-κB pathway for the therapy of diseases: mechanism and clinical study. Signal Transduct Target Ther.

[26] Jung SW, Moon JY (2021). The role of inflammation in diabetic kidney disease. Korean J Intern Med.

[27] Chen CY, Chang JT, Ho YF, Shyu AB (2016). MiR-26 down-regulates TNF-α/NF-κB signalling and IL-6 expression by silencing HMGA1 and MALT1. Nucleic Acids Res.

